# Researches upon the Necropolis of New Orleans

**Published:** 1851-01

**Authors:** 


### ARTICLE III.

*Researches upon the Necropolis of New Orleans, with brief allusions to its vital Arithmetic.* By BENNETT DOWLER, M. D. &c.

Dr. Dowler is noted for his patient, industrious researches, and the present pamphlet is evidence that there is at least one physician whose conscience does not warn his feet from the cemetery. The pamphlet before us is written in defence of the salubrity of New Orleans, concerning which, the Dr. assures us, people have very erroneous notions. That the rate of mortality is excessively great in that city, he does not pretend to deny; but his inspections of the epitaphs in the cemeteries, go to show that the great destruction of life is amongst emigrants aud other unacclimated persons, while the native and acclimated population attain to as great an age as elsewhere. He makes out a pretty strong case, and we are pleased to learn that the "Wet Grave-yard" is not such a terrible place as it has been represented.

While we are disposed to commend Dr. Dowler for the enthusiasm and industry which he has so abundantly displayed in the cause of Medical science, we regret to notice that his articles are so obnoxious to literary critcism. The style is at times labored, and frequently obscure, with a tendency to digression: there is also a fondness for the use of *outre* phrases, which are a serious blemish to a style which in the main possesses much vivacity. M.

